# Mitochondria, immunosenescence and inflammaging: a role for mitokines?

**DOI:** 10.1007/s00281-020-00813-0

**Published:** 2020-08-05

**Authors:** Maria Conte, Morena Martucci, Antonio Chiariello, Claudio Franceschi, Stefano Salvioli

**Affiliations:** 1 Department of Experimental, Diagnostic and Specialty Medicine (DIMES), University of Bologna, Bologna, Italy; 2 Interdepartmental Center “Alma Mater Research Institute on Global Challenges and Climate Change (Alma Climate)”, University of Bologna, Bologna, Italy; 3 Laboratory of Systems Medicine of Healthy Aging and Department of Applied Mathematics, Lobachevsky University, Nizhny Novgorod, Russia

**Keywords:** Mitochondrial metabolism, Mitokines, Inflammaging, Immunosenescence, Human ageing

## Abstract

A global reshaping of the immune responses occurs with ageing, indicated as immunosenescence, where mitochondria and mitochondrial metabolism play an important role. However, much less is known about the role of mitochondrial stress response in this reshaping and in particular of the molecules induced by such response, collectively indicated as mitokines. In this review, we summarize the current knowledge on the role of mitokines in modulating immune response and inflammation focusing on GDF15, FGF21 and humanin and their possible involvement in the chronic age-related low-grade inflammation dubbed inflammaging. Although many aspects of their biology are still controversial, available data suggest that these mitokines have an anti-inflammatory role and increase with age. Therefore, we hypothesize that they can be considered part of an adaptive and integrated immune-metabolic mechanism activated by mitochondrial dysfunction that acts within the framework of a larger anti-inflammatory network aimed at controlling both acute inflammation and inflammaging.

## Introduction

During ageing, an imbalance between inflammatory and specific immune responses occurs, leading to a decreased efficiency of these latter. It is known in fact that elderly people are more susceptible to infectious diseases and have a lower response to vaccination, with respect to young people. In particular, an increased inflammatory response blocks the activation of B and T cell responses [1–6]. This phenomenon, indicated as “immune paralysis” [7] or sometimes “immune activation” [8], can be considered one of the most striking clinical features of immunosenescence, and it is at least in part due to an excess activation of immune cells, mediated by pro-inflammatory cytokines [9–14]. B cells from both mice and humans are also impaired as a result of exposure to pro-inflammatory cytokines such as TNF-α [15, 16]. In fact, high levels of TNF-α induce a significant decrease in the capacity of B cells to produce protective antibodies [15, 16]. Other cell types like macrophages and NK cells undergo immune paralysis after a prolonged inflammation. To this regard, it has been reported that human monocytes display phagocytosis defects up to 6 months after a recovery from an inflammatory condition [17]. As far as NK cells, a decrease of NK cell number and functionality has been reported in experimental sepsis [18]. Moreover, it is also reported that chronic inflammation can drive the induction of myeloid-derived suppressor cells (MDSCs), which in turn create a microenvironment favourable to immune suppression [19–21]. To this regard, an almost universal phenomenon occurring during ageing is a state of chronic, low-grade, sterile inflammation that has been termed inflammaging [22]. Since many years, the role of energy metabolism in immune responses and inflammation is acknowledged (see below). However, the picture is much less clear as far as the role of mitochondrial stress response in these phenomena. In fact, it is known that a series of reactions are elicited in response to a mitochondrial dysfunction. In this short review, we will focus on the possible involvement of mitokines, i.e. soluble molecules produced in response to mitochondrial stress, in immunosenescence and inflammaging.

Inflammaging has long been considered a feature of immunosenescence, as it was thought that macrophages were the main responsible for the observed age-related increase of soluble inflammatory mediators [22]. Since then, the role of other cells and tissues as well as the contribution of other biological processes such as meta-flammation and cellular senescence has been recognized; however, the importance of immune cells in inflammaging is likely not negligible, considering in particular the capability of immune cells to recognize self-molecules such as uric acid crystals, heat shock proteins and mitochondrial components. These latter include cardiolipin, N-formyl peptides, TFAM transcription factor, mitochondrial DNA (mtDNA) and double-stranded RNA (dsRNA) [23, 24]. These self-molecules, when misplaced or altered in any way, can be sensed by macrophages through innate immunity receptors and can induce the production and release of pro- and anti-inflammatory mediators such as IL-6, TNF-α, IL-1β, RANTES and IL-1ra [25–28]. Collectively, these self-molecules are indicated as DAMPs (danger-associated molecular patterns), and their receptors are indicated as PRR (pattern recognition receptors). More recently, the pro-inflammatory role of at least some of those molecules has been extended also to non-immune cells, as they can be sensed also through widely expressed intracellular receptors, such as inflammasomes, mitochondrial antiviral signalling (MAVS) proteins, cGAS-Sting and toll-like receptor 9 (TLR 9), leading to a robust inflammatory and type I interferon response [24]. We have hypothesized that a possible cue for understanding inflammaging resides in the age-dependent increment in the production (or inefficient clearance) of these DAMPs, a phenomenon that we proposed to indicate as “garb-aging” [29]. As a solid example of garb-aging, we have reported that circulating levels of mtDNA increase with age and that people with highest levels of circulating mtDNA show higher concentrations of IL-6, TNF-a, RANTES and IL-1ra with respect to those with the lowest levels of mtDNA [30].

Other than sources of DAMPs, mitochondria are important modulator of immune responses also because of their role in energy metabolism, as it is known that T lymphocyte activation entails a shift from oxidative phosphorylation (OXPHOS) to aerobic glycolysis [31], though OXPHOS is still present and needed for the functional differentiation. In particular, it has been reported that succinate dehydrogenase is needed for T cell differentiation into Th1, while OXPHOS complex I, the malate-aspartate shuttle and the mitochondrial citrate export are required for proliferation [32]. Considering that one of the key features of ageing appears to be mitochondrial dysfunction, it is more than likely that such dysfunction can also impinge upon proper T cell function [23]. Accordingly, it has been recently reported that CD4+ T cells from elderly people are characterized by the presence of an elevated number of dysfunctional mitochondria engulfed into autophagosomes with respect to cells from young people, suggesting the presence of a defective mitochondrial turnover. These defective mitochondria may be the source of inflammatory stimuli, as mentioned above, and contribute to the impairment of immune defences in older people [33–35].

As far as B lymphocytes, it is reported that activation of naïve B lymphocytes leads to an increase in mitochondrial mass and number as well as expression of genes for TCA cycle and OXPHOS [36] and, on the other side, inhibition of OXPHOS (by oligomycin) or TCA (by glutamine depletion) blocks the activation of B cells and differentiation into plasmablasts, as well as proliferation and antibody class switching [36, 37]. These data suggest that also for B cells mitochondrial energy metabolism is important and that mitochondrial dysfunction can be at the basis of B cell functional impairment.

Finally, it is known that a main feature of the ageing bone marrow is a shift toward the production of myeloid cells at the expenses of lymphoid ones [38], and, consistently, it has been found that in a mouse model of mitochondrial impairment (UCP2 knockout, leading to an increased production of ROS), aged animals display an increased amount of monocytes and neutrophils and a decreased amount of B cells [39].

## Mitochondrial stress response and immune responses

Despite the fact that, as mentioned in the previous paragraph, mitochondrial dysfunction can be a cause of immune response impairment, it has been proposed that a mild mitochondrial dysfunction could be beneficial for the cell as it could elicit an effective stress response [40]. This hypothesis was introduced a few years ago by Johnson and co-workers in a study conducted on *Caenorhabditis elegans*. They demonstrated that a mild disruption of mitochondrial electron transport chain (ETC) causes a transient DNA (nuclear and mitochondrial) damage leading to the activation of compensatory mechanisms that increase the lifespan in *C. elegans* [41]. A detailed discussion on the retrograde stress responses elicited by a mitochondrial stress, such as the mitochondrial unfolded protein response, is outside the scope of this short review, as many excellent studies have been published on this topic [40, 42, 43]. Rather, we will focus on the effects of some downstream products of this stress response on the immune function and on inflammation.

In their 2011 seminal paper, Dillin and co-workers demonstrated that the beneficial effect of a mild mitochondrial stress is not limited to the affected cell/tissue but also spreads to distal ones, thus conferring a global resistance and survival advantage to the whole organism [44]. In this paper, the authors presented evidence that an impairment of the electron transport chain localized in one tissue can be perceived in distal ones. In other words, the signalling of a localized mitochondrial perturbation can actually spread through the organism via soluble mediators that these authors indicated as “mitokines”. A mitokine should be a soluble molecule (protein, peptide or other) produced and secreted in response to a mitochondrial stress response and able to elicit an adaptive/compensatory response in distal cells even though not directly affected by the stressful event/stimulus. Since then, a number of molecules have been identified that fulfil this definition, including the neuronal peptide FLP2 in *C. elegans* [45], the fibroblast growth factor 21 (FGF21) [46–48] and the growth differentiation factor 15 (GDF15) [49, 50] and a series of mitochondrial DNA-encoded peptides that include humanin (HN) [51], MOTS-c [52, 53] and small humanin-like peptides (SHLPs) [54]. We have reported that some of them, namely, GDF15, FGF21 and HN, increase with age from young people to centenarians and are associated with worsened haematochemical parameters and, for nonagenarians and centenarians, with lower life expectancy [55]. Consistently, GDF15 has been reported to be the most upregulated protein in old age [56, 57] associated with many pathological conditions, such as type 2 diabetes (T2D), cardiovascular diseases (CVD), neurodegeneration and overall mortality [50, 58–60]. FGF21 is considered a pro-longevity hormone as it is able to modulate energy and lipid metabolism and extend animal lifespan [46, 47, 61–63]; however, it is also involved in accelerated ageing and premature death in Opa-1-deficient mice [64]. HN has been identified as a powerful anti-apoptotic factor with a cytoprotective role in many age-associated diseases such as Alzheimer’s disease (AD), T2D and CVD [65]. As a whole, these data support the idea of mitohormesis proposed by Ristow and Zarse [66]. According to this idea, the pro-longevity effects of some treatments like calorie restriction are ultimately due to an increased formation of ROS, which in turn activates a retrograde response of stress resistance culminating in lifespan extension. The observed age-related increase of mitokines could be then considered an attempt of mitohormesis aimed at increasing stress resistance [55, 67]; however, when the production of otherwise beneficial mitokines turns from acute to chronic (as it occurs with age), it becomes either no longer effective or even toxic [55]. In the next paragraphs, we will briefly summarize the present knowledge on the involvement of mitokines in the ageing of the immune system (immunosenescence), with particular regard to inflammaging.

## Mitokines in immune responses

The role in immune response and inflammation of the mostly studied mitokines will be briefly described below, and, where available, data on ageing will be discussed.

### GDF15

GDF15 was discovered over 20 years ago as a member of the transforming growth factor β (TGF-β) superfamily. The function of GDF15 is not still fully clear, since several findings propose opposite roles for this protein. While on one hand many studies suggest a protective/anti-inflammatory role for GDF15, on the other hand, many studies hypothesize a pathogenic/pro-inflammatory function.

Several studies in humans have reported that GDF15 activity increases under stress conditions in response to tissue insults. In healthy individuals, as well as young subjects, the circulating concentration of GDF15 is very low. Conversely, plasma GDF15 levels are higher in the elderly, in particular in the presence of pathological conditions, such as CVD, insulin resistance and T2D, neurodegeneration, renal chronic disease and cancer [50, 55], where it is supposed that GDF15 plays a protective role against different insults via PI3K–Akt, ERK1/2 and SMAD2/3 signalling pathways [68–71]. Recently, it has been shown that GDF15 decreases the expression of pro-inflammatory cytokines and prevents the activation of T cells in the liver of mice with fibrosis, while deficiency of GDF15 aggravates liver injury and fibrosis [72]. In agreement, Bootcov and co-workers found that the expression of GDF15 is induced in macrophages by IL-1β, IL-2, TNF-α and TGF-β and limits their activation, consequently blunting inflammation [73]. GDF15 is responsive to inflammation via p53 [74] and is necessary for tolerance to inflammation induced by viral or bacterial infections [75]. We have reported that GDF15 in humans is associated with increased number of total leukocytes and decreased number of lymphocytes [76]. This is also in agreement with a previous report showing an increase of GDF15 and a concomitant decrease in circulating CD4+ and CD8+ T cells in patients with COPD [77]. Therefore, all these findings suggest that GDF15 may have an anti-inflammatory role, although there are also studies suggesting the opposite. In particular, a study in mouse models indicates that the deletion of haematopoietic GDF15 reduces CCR2 expression and chemotaxis and improves plaque stability, with beneficial effects against atherosclerosis [78]. In agreement, another study in mice demonstrates that GDF15 is involved in the progression of atherosclerosis by regulating apoptotic cell death and IL-6 inflammatory response [79]. The hypothesis that GDF15 may play detrimental role is also supported by many studies on the effects of GDF15 on cachexia. Higher circulating GDF15 levels in fact were found to be associated with the development of cachexia in both animal models and human patients [80–82]. In particular, gain- and loss-of-function experiments in mouse models suggest that GDF15 is a mediator of cancer cachexia [81]. In addition, GDF15 is also considered a marker of all-cause mortality [50, 83], and in agreement in our previous study, we found that, among mitokines, GDF15 is the most associated with mortality in old age [55]. Therefore, the role of GDF15 is more complex than expected and possibly double-sided. In a condition of mild or transient stress (or when a pathology is in an early stage), the increase in circulating GDF15 levels could be protective; on the contrary, when the stress is elevated and chronic (or when a pathology is in an advanced stage), the continuous/chronic release of GDF15 may become detrimental [55, 84]. It is also possible that, very simply, in chronic situations, the detrimental effects that caused GDF15 expression overcome GDF15’s beneficial ones. Interestingly, GDF15 appears the most upregulated protein during the ageing process, as mentioned above [56, 57]; therefore, its importance is probably higher than expected and plays a crucial role in the interconnection between metabolism, inflammation and immune response in old age.

Given the large number of actions associated with GDF15, a still unresolved question is how GDF15 acts and by what type of receptors. To date, the only confirmed GDF15 receptor is the GDNF receptor family member GFRAL, acting with its co-receptor RET. However, several studies in mice, as well as in nonhuman primates, reported that GFRAL expression is present only in hindbrain neurons but not in other peripheral tissues [85, 86]; therefore, it is not yet clear how GDF15 can act directly on peripheral tissues in the absence of any known receptors. It is then possible that still not recognized receptors exist other than GFRAL or that GDF15 may exerts its effects through other molecules. Actually, previous studies showed that GDF15 might act on different cell types through TGF-β and its receptors, as GDF15 is involved in the regulation of TGF-β/Smad signalling pathway [74, 87–90]. However, these findings must be interpreted with caution, as it has been reported that commercial preparations of recombinant GDF15 can be contaminated with variable levels of TGF-β [91].

### HN

HN plays a cytoprotective role against oxidative stress, apoptosis and inflammatory response [65]. A role for HN in immune responses has been, in fact, demonstrated in several studies, suggesting that HN has a role in the attenuation of inflammation and it has therefore to be considered an anti-inflammatory mediator [92–95]. In particular, the neuroprotective role of HN has been at least in part attributed to the capability of HN to interact with the gp130 subunit of the IL-6 receptor, leading to a decreased in vitro production of pro-inflammatory cytokines, such as IL-6, IL-1β and TNFα [92, 94]. HN attenuates inflammation and macrophages infiltration, as well as apoptosis, also in the early stage of kidney disease in ApoE-deficient mice [93]. Moreover, it is possible that the anti-inflammatory activity is partly mediated by the well-known anti-apoptotic effects of HN, as it can dampen the production of pro-inflammatory, apoptosis-related DAMPs. To mention only few examples of the anti-apoptotic activities of HN, it has been demonstrated that HN physically interacts with IGFBP-3, a pro-apoptotic protein [96]. In particular, IGFBP-3 expression is very elevated in brain affected by AD suggesting a role for IGFBP-3 in cell death [97]. Moreover, it has been demonstrated that HN interacts with the apoptosis-inducing protein Bax, creating a HN-Bax complex. In this way, HN retains Bax in the cytoplasm and prevents the translocation of Bax to mitochondria, thus protecting from apoptosis [98]. This activity is important also for inflammation, as the activation of Bax/Bak complexes leads not only to the release of pro-apoptotic molecules but also of mtDNA and dsRNA that can activate a pro-inflammatory response via MAVS, cGAS-Sting, inflammasomes and NF-κB [24]. Consistently with the idea that HN has not only cytoprotective but also anti-inflammatory roles, it has been found that HN has protective roles not only in AD [94] but also in T2D, CVD and atherosclerosis [95, 99–101], which are all considered inflammaging-related diseases [102, 103]. Moreover, very recently it has been reported that HN, and probably other mitochondria-derived peptides, improves not only health status but also lifespan [104]. In particular, Yen and co-workers found that the overexpression of HN increases lifespan in *C. elegans*, while middle-aged mice treated with the potent humanin analogue HNG showed an improvement in health parameters [104]. Literature data are still controversial as far as the age-related changes in HN circulating levels. At variance with some studies that showed a decrease of HN with ageing in both mice and humans, we have found in humans an increase of HN with age. To further support this finding, we have also observed that higher levels of HN and GDF15 are present in people with accelerated ageing such as Down Syndrome (DS) persons, with respect to their siblings of similar age [55]. In agreement with our finding, a recent work by Salemi et al. [105] shows a significant upregulation of HN in fibroblasts from DS persons compared with non-trisomic siblings [105]. As a whole, it is tempting to speculate that these increases in HN concentrations can be considered an attempt of the tissue/organism to counteract the detrimental effects of inflammation/inflammaging.

### FGF21

FGF21 is an important regulatory protein of energy metabolism and inflammatory processes. Several studies of gain- and loss-of-function indicate that FGF21 is a key metabolic mediator to improve the compromised mitochondrial function and reduce inflammation and apoptosis in several organs [106–108]. An in vitro study in LPS-stimulated murine macrophagic cells (RAW 264.7) demonstrated that a pre-incubation with FGF21 reduces the expression of the pro-inflammatory cytokines TNF-α, IL-1β and IL-6, increases the level of IL-10 and inhibits the activation of the nuclear factor-κB (NF-κB) [109]. This finding was then confirmed and further investigated by many other studies. For example, a recent study from Gao et al. [110] demonstrated that FGF21 suppressed inflammation and apoptosis caused by LPS stimulation via inhibition of TLR4/MYD88/NF-κB signalling pathway in both Balb/c mice and BEAS-2B or THP-1 cells [110]. Moreover, in a rat model of atherosclerosis, the upregulation of FGF21 affected inflammation and oxidative stress by increasing the expression of Nrf2-ARE (nuclear factor erythroid 2-related factor 2-antioxidant response elements) signalling-related proteins [111], which are involved in cellular antioxidant and anti-inflammatory pathways, as well as in the protection of mitochondria.

In addition to its anti-inflammatory role, FGF21 seems to play also an important protective role against thymus ageing by delaying age-related thymic involution in mice [112]. In particular, the overexpression of FGF21 reduces the generation of ectopic lipids and inflammation, while its loss-of-function in middle-aged mice accelerates thymic involution. Therefore, it seems that FGF21 can be considered a key regulator of the immune function able to counteract at least in part the immune dysfunction occurring with ageing. In agreement with these positive roles in immune function, FGF21 has a pro-longevity activity in mice [93] and is considered a marker of successful ageing [113]. However, the precise role of FGF21 in ageing is still highly questioned. In fact, it is reported that FGF21 could be responsible for the accelerated ageing phenotype observed in Opa-1 KO mice [64], and consistently, in our previous study, we have found that circulating FGF21 levels increase with age in subjects without evident pathologies and are particularly higher in centenarians. Moreover, we have found that FGF21 is positively associated with worsened parameters, such as higher insulinemia and HOMA-IR, and is inversely correlated with survival in oldest subjects [55]. Therefore, whether the role of FGF21 in ageing is beneficial or detrimental is still to be clarified. It has to be considered that, similarly to GDF15, an acute or chronic production of FGF21 may have different or even opposite biological meanings. Therefore, it could be speculated that an acute increment of FGF21 is good for health and lifespan, while a chronically elevated level of circulating FGF21 could be detrimental or, more likely, could be interpreted as an attempt of the organism to counteract an overwhelming stress.

### Other mitokines

Much less is known regarding the role in immune responses/inflammation of mitokines such as FLP2, MOTS-c and SHLPs.

FLP2 neuropeptide is important for the regulation of mitochondrial stress response in the nervous system of *C. elegans*. FLP2 is released by neurons during mitochondrial dysfunction and transmits to distal tissues a signal for the induction of UPR^mt^ [45]. However, its overexpression does not extend worms’ lifespan [45]. No data are available regarding the possible involvement of FLP2 in the ageing process, as well as inflammation or immune response. However, since many studies indicate that UPR^mt^ affects inflammatory responses [114–117], it is possible that, at least indirectly, FLP2 can have a role as well.

MOTS-c is a peptide of 16 amino acids discovered few years ago by Lee et al. [53]. MOTS-c is expressed in several tissues, such as skeletal muscle and adipose tissue, but it is also present at circulating level, both in mice and humans [53, 118]. Studies suggested that MOTS-c is a key regulator of cellular metabolism and inflammatory processes [53, 119]. It has been shown that MOTS-c counteracts inflammation by reducing the levels of pro-inflammatory cytokines, such as IL-6, IL-1β and TNFα, and increasing those of the anti-inflammatory cytokine IL-10 [53, 120]. In particular, MOTS-c decreases the bacterial load in mice with sepsis by enhancing the bactericidal capacity of macrophages and thus improving the survival of mice [120]. Moreover, MOTS-c suppresses inflammation also by controlling NF-κB and STAT1 pathway [121]. As far ageing, it seems that MOTS-c circulating levels decrease with ageing [53, 122].

SHLPs are a class of peptides (from 1 to 6) expressed in several tissues and detectable at plasma level [54]. To date little is known about their precise role; however, in vitro studies demonstrated that they have a number of biological effects. In particular, SHLP2 and SHLP3 increase cell viability and reduce apoptosis, while SHLP6 significantly enhances apoptosis [54]. Similar to HN, SHLP2 and SHLP3 play a critical role in metabolism, apoptosis and inflammation [54]. The mechanism by which SHLPs regulate the expression of metabolic and inflammatory markers remains still unclear; however, data from Cobb et al. suggest that SHLP2 and SHLP3 regulate the levels of leptin, while SHLP3 regulates also those of IL-6 and MCP-1 [54]. In addition, SHLP2 treatment protects against Aβ1–42-induced cell death and thus can counteract AD [54]. Moreover, SHLP2 circulating levels decline with age. Altogether, these data suggest that SHLP2 and SHLP3 have potential beneficial effects and can counteract (at least some) age-related diseases [54, 123, 124].

## Conclusions and perspectives

**Fig. 1:**
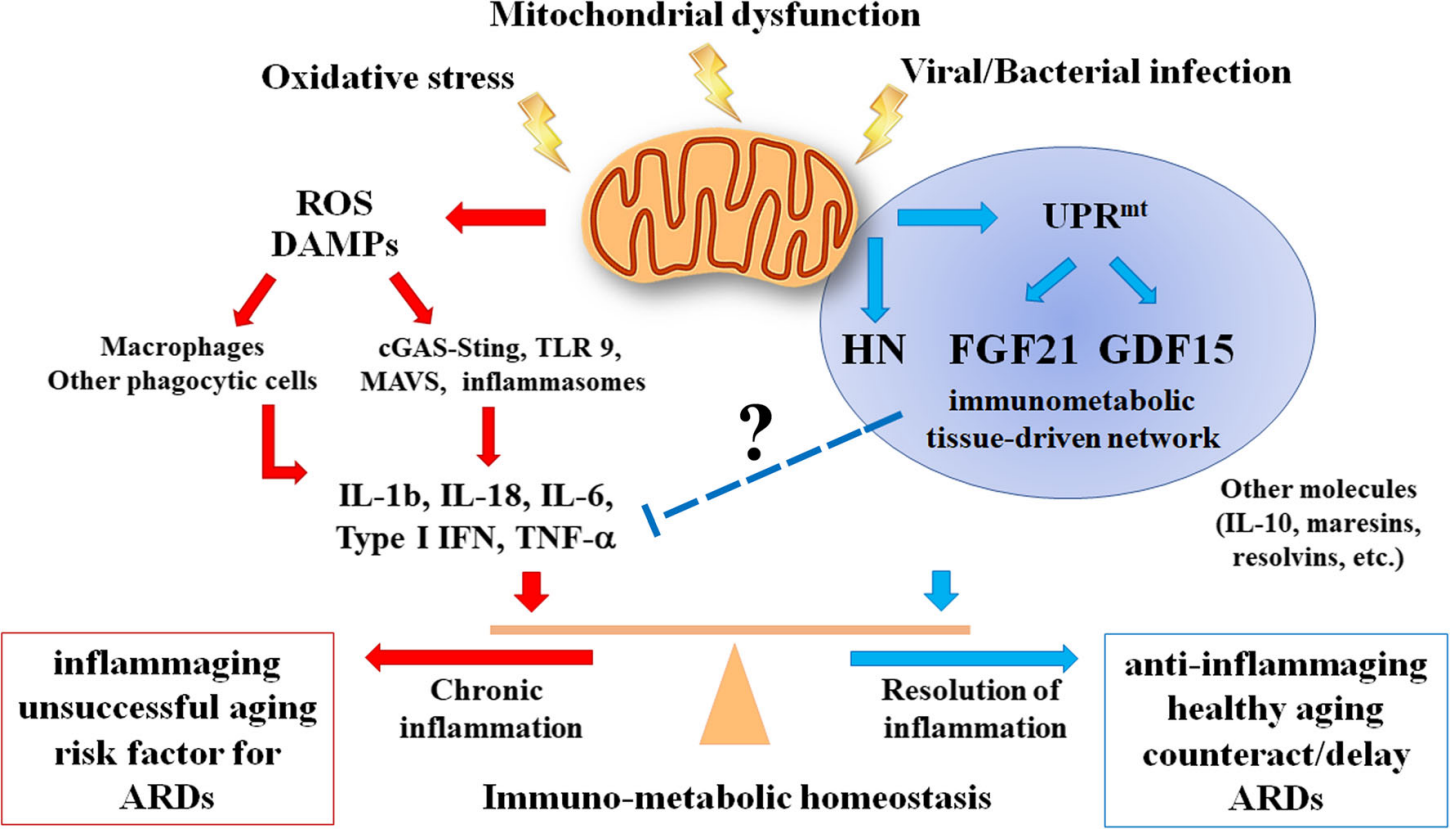
Schematic representation of the role of mitochondria in healthy ageing or unsuccessful ageing and the onset of age-related diseases (ARDs). In healthy ageing, stress stimulates the mitochondrial unfolded protein response (UPRmt) and the production of mitokines (HN, FGF21, GDF15) that act to inhibit production and activity of inflammatory cytokines (including IL-1β, IL-18, IL-6, type I IFN, TNF-α) through yet not clarified mechanisms and thus preserve a balance between inflammatory and specific immune responses; in unsuccessful ageing, an imbalance between inflammatory and specific immune responses occurs, with a high production of reactive oxygen species (ROS) and danger-associated molecular patterns (DAMPs) leading to an increase of inflammatory cytokines that contribute to the onset of ARDs. GDF15, FGF21 and HN can be part of an immune-metabolic machinery/network activated by both acute and chronic stressors impinging on mitochondria aimed at modulating inflammaging. Many other anti-inflammatory molecules are known, including IL-10, resolvins, maresins, etc

Even if the role of mitochondrial stress response in modulating the immune responses is only partially understood, available data point out to an anti-inflammatory role of the most studied mitokines. As summarized in the previous paragraphs, GDF15, FGF21 and HN have been proposed as anti-inflammatory molecules in many experimental conditions, in both humans and animal models. As their blood levels appear to increase with age (as many pro-inflammatory mediators do), it is possible that they are part of an integrated, molecular, immune-metabolic machinery/network able to respond to (among others) mitochondrial stress, whose role is to set up a coordinated, systemic anti-inflammatory response in both acute and chronic conditions. In this latter case, the mitokine response could be considered part of an “anti-inflammaging” process whose existence was proposed some years ago as a consequence of inflammaging [124]. In fact, the capability to downregulate both acute and chronic inflammatory responses is crucial to maintain homeostasis in young subjects and to avoid/postpone age-related diseases (CVD, T2D, neuro-inflammation and cancer, among others) in the elderly. Many other anti-inflammatory molecules are known, including resolvins, maresins, adiponectin, IL-10, TGF-β, etc. Some of them are particularly elevated in centenarians [125–128], suggesting that these exceptional people owe their longevity, among others, to a successful balancing between pro- and anti-inflammatory mediators. Now, the discovery that other molecules endowed with anti-inflammatory and immunomodulatory activity increase with age not only in centenarians but also in elderly people suggests that a large, comprehensive anti-inflammaging network is physiologically put in place likely as a general attempt of the ageing organism to cope with the progressive increase of chronic inflammation (inflammaging) and its deleterious effects. However, ultimately this (beneficial) response can be insufficient to counteract the detrimental accumulation with age of molecular insults, particularly in those old people affected by major are-related pathologies. Thus, the increased blood levels of mitokines such as GDF15, FGF21 and HN become correlated with altered haematochemical parameters and lower life expectancy [55]. A simplified version of this hypothesis is presented in Fig. 1. The key concept expressed in the figure is the balancing between pro- and anti-inflammatory stimuli that are tightly interconnected via feedback loops to produce a net result of detrimental or successful adaptation to a chronic stress. The other key concept is that the source of the chronic stress is not necessarily an external *noxa*, but it can be the inescapable, progressive process of deterioration of crucial organelles such as mitochondria. Thus, all the systems aimed at sensing and responding to mitochondrial dysfunction occupy the centre of the stage for effective adaptation/remodelling of the different cell types and organs involved, leading eventually to either successful or unsuccessful ageing. As far as immune responses other than inflammation, the precise role of mitochondrial stress response and mitokines is less clear, and more studies are needed to better clarify this topic, in particular in the framework of immunosenescence and of the crosstalk between the immune system and other organs such as the liver and adipose tissue. As other types of stresses, like ER stress, can also elicit the production of some mitokines, future studies should disentangle the precise role of mitochondrial dysfunction with respect to other stresses in modulating the immune responses via mitokine production. Moreover, studies aimed at better clarifying the direct responsiveness of mitokines to inflammatory or immunological stimuli would be also desirable. However, should the hypothesis that mitokines are part of an attempt to modulate acute and chronic inflammatory reactions be confirmed, a reappraisal of the biological meaning of their association with morbidity (age-related diseases) and mortality, is urgently needed, also considering their possible use (positive modulation of their expression) as therapeutic tools and targets.
